# Clinicopathological Analysis of a Group of Patients Diagnosed with Endometrial Cancer and Mutation in the Tp53 Gene—Single-Center Study

**DOI:** 10.3390/jcm14196809

**Published:** 2025-09-26

**Authors:** Dominik Jakubowski, Aleksandra Kukla-Jakubowska, Kaja Michalczyk, Marcin Misiek, Janusz Menkiszak, Anita Chudecka-Głaz

**Affiliations:** 1 Department of Diagnostic Imaging and Interventional Radiology, Pomeranian Medical University in Szczecin, 71-252 Szczecin, Poland; 2 Department of Gynecological Surgery and Gynecological Oncology of Adults and Adolescents, Pomeranian Medical University, 70-111 Szczecin, Poland; 3 Department of Gynecologic Oncology, Holy Cross Cancer Center, 25-734 Kielce, Poland

**Keywords:** endometrial cancer, TP53-mutation, FIGO-staging, lymphovascular space invasion

## Abstract

**Background/Objectives**: Endometrial cancer (EC) remains a significant clinical challenge due to increasing incidence and mortality, particularly among patients with TP53 gene mutations, which define a high-risk molecular subtype. This study aimed to characterize the clinicopathological and molecular features of a cohort of patients diagnosed with endometrial cancer and confirmed TP53 mutations. **Methods**: This retrospective single-center study analyzed 20 patients with histologically confirmed EC and pathogenic TP53 mutations treated at the Pomeranian Medical University Clinical Hospital No. 2 between January 2023 and March 2025. Clinical, histological, and molecular data—including FIGO stage, tumor grade, and coexisting mutations—were collected. **Results**: Patients had a mean age of 69.2 years and a mean BMI of 29.5 kg/m^2^. The most common histological types were endometrioid (45%) and serous carcinoma (40%). Grade 3 tumors were found in 65% of cases, and 65% of patients exhibited lymphovascular space invasion. Notably, 30% of patients were upstaged under the FIGO 2023 classification when incorporating TP53 mutation status. Four patients had coexisting PIK3CA mutations. No significant differences were observed in BMI, endometrial thickness, or abnormal bleeding between histological subgroups. **Conclusions**: TP53-mutated endometrial cancers are associated with aggressive histopathological features and advanced staging. Molecular profiling, particularly TP53 mutation assessment, provides essential prognostic information and may inform personalized therapeutic strategies. Larger, multicenter studies are warranted to validate these findings and identify actionable molecular targets.

## 1. Introduction

Endometrial cancer is one of the most common gynecological cancers in developed countries [1]. It is the sixth most frequently diagnosed cancer in women worldwide [2]. There has been a steady increase in diagnosed cases in recent years, making endometrial cancer a serious health problem. Despite advances in treatment, cancer-related mortality has increased in some countries over the past decade [3].

A breakthrough in endometrial cancer diagnosis was the introduction of molecular classification by The Cancer Genome Atlas (TCGA). Genomic analysis allowed for the identification of four molecular subtypes, which have a higher prognostic value than histopathological evaluation alone [4]. These subtypes are as follows:

(1) POLE-ultramutated (POLEmut): This is characterized by a very high number of mutations and an excellent prognosis [5].
(2) Microsatellite Instability-High (MSI-H)/Mismatch Repair Deficient (dMMR): This type is associated with microsatellite instability, a high mutation rate and intermediate prognosis.
(3) Copy Number Low (CN-L)/No Specific Molecular Profile (NSMP): This type has a low number of mutations and an intermediate prognosis.
(4) Copy Number High (CN-H)/Serous-like: This subtype is characterized by a high level of chromosomal instability, a low overall mutation count, common TP53 mutations, and the worst prognosis of all subtypes.

The TP53 gene encodes a p53 protein, which plays a key role in maintaining genome stability. In response to a variety of factors such as DNA damage, hypoxia or oncogene activation, the p53 protein becomes activated and acts as a transcription factor. It regulates the expression of genes involved in cell cycle control, DNA repair and the initiation of apoptosis. Mutations in the TP53 gene are the most common genetic alterations in cancer, being found in over 50% of cases [6]. For endometrial cancer, this mutation is present in approximately 25% of patients overall, with a much higher frequency in type II groups (approx. 90%) than type I (10–40%). The literature highlights the fact that mutated forms of the p53 protein not only lose their original functions, but also acquire oncogenic properties, promoting neoplastic cell proliferation, invasiveness and resistance to treatment. Mutation of the TP53 gene covers a wide spectrum of alterations—the most common are missense mutations, resulting from a single nucleotide substitution leading to a change in one amino acid in the protein (they account for approx. 70–80% of mutations) [7]. Moreover, nonsense mutations, frameshifts, deletions and insertions can also occur.

Studies to date have emphasized the association of TP53 gene mutations (and often abnormal p53 expression, as assessed by immunohistochemistry) with the well-established risk factors for endometrial cancer. Patients with this molecular type of endometrial cancer more often present with an advanced stage (according to the FIGO classification, stage III or IV) at the time of diagnosis [8]. Correlations between a higher degree of histological malignancy (grading) and belonging to histological types other than endometrioid carcinoma have also been noted [9]. Furthermore, TP53 mutations have been associated with deep myometrial invasion, frequent cervical stromal invasion, adnexal involvement, and lymphovascular space invasion (LVSI) [7]. Numerous studies confirm that TP53 mutation status (or abnormal p53 protein expression) is one of the strongest predictors of an unfavorable prognosis in endometrial cancer [4]. Patients with p53-abnormal tumors have significantly shorter overall (OS) and disease-free (DFS) survival compared to patients with p53-wildtype tumors. Therefore, TP53 status has been proven to provide additional prognostic information beyond that resulting from stage or histological type alone. The current scientific consensus recognizes TP53 mutation as a key independent marker of poor prognosis in patients with endometrial cancer; its prognostic value remains independent of other clinical factors, as confirmed by multivariate analyses [10].

The increasing role of molecular testing in patients diagnosed with endometrial cancer was highlighted in the FIGO 2023 classification, where, for the first time, the results of molecular classification were directly incorporated into the staging assessment [11]. Currently, staging is determined hierarchically: first, clinicopathological features are assessed (analogous to the FIGO 2009 classification, but taking into account new rules regarding LVSI); then, stage is adjusted based on molecular type. When there is a tumor confined to the uterus (irrespective of the depth of myometrial infiltration), patients with a POLEmut mutation are classified as stage IA, a downstaging compared to the previous classification, according to which the stage could be defined as IB. This difference reflects the excellent prognosis of patients with a mutation in the POLE gene. Patients with abnormal expression of p53 protein or TP53 mutations are often upstaged; for example, in FIGO I and II, patients in whom endometrial cancer is confined to the endometrium with any invasion of the uterine muscle, with or without the invasion of the cervix, regardless of the LVSI grade and histological type, will be determined as stage IICmp53abn.

## 2. Materials and Methods

The study included 20 patients with histopathologically diagnosed endometrial cancer and a confirmed mutation in the TP53 gene, who were hospitalized at the PUM [Pomeranian Medical University] University Clinical Hospital No. 2 in Szczecin between January 2023 and March 2025. All procedures adhered to the principles outlined in the Declaration of Helsinki. Patient informed consent was waived for this study due to its exclusively retrospective design. All clinical and molecular data used in the analysis were fully anonymized and de-identified prior to access by the research team. This process ensured that patient confidentiality was maintained and made it impossible to trace data back to individual patients, thereby posing no risk to the individuals whose records were included. Inclusion criteria: histopathologically confirmed diagnosis of endometrial cancer, no previous oncological treatment, and known mutation in the TP53 gene. Exclusion criteria: concomitant presence of other neoplasms, coexistence of advanced renal or heart failure and incomplete histopathological data.

Patients were classified into two groups depending on the histopathological type of tumor: Group I included patients diagnosed with an endometrial glandular tumor and Group II included patients with other histological types. Qualitative variables included age, number of pregnancies and deliveries, history of abnormal vaginal bleeding, use of menopausal hormone therapy and smoking status. The quantitative variable was endometrial thickness, which was assessed by transvaginal ultrasound (TVUS) upon hospital admission. All assessments were performed by a physician with at least five years of experience in gynecological ultrasound to ensure consistency. To standardize the procedure, a specific measurement protocol was followed: endometrial thickness was measured in the long-axis, mid-sagittal view of the uterus. The measurement was taken at the thickest point, encompassing the full anteroposterior diameter of the endometrial echo from the anterior to the posterior endometrial-myometrial interface. Any intracavitary fluid was carefully excluded from this measurement.

The analysis took into account the tumor’s histological type, stage and LVSI status, as well as the FIGO stage. Tumor fragments or formalin-fixed, formalin-embedded slices (PFFE) constituted biological material used for the study. The molecular study was carried out with next-generation sequencing (NGS), using an Illumina MiSeq sequencer; the VariantPlex VP endometrial panel kit from Archer Dx was used to prepare the library. The scope of the study included all coding exons of the EPCAM genes, i.e., MLH1, MSH2, MSH6, PIK3CA, PMS2, POLE and TP53, with a surrounding intron sequence of up to 30 base pairs.

Anthropometric measurements, including weight (kg), height (cm) and waist and hip circumferences (cm), were taken during hospitalization. Body mass index (BMI) was calculated using the formula of body weight (kg)/height (m^2^).

Continuous variables were summarized as the mean ± standard deviation or median and range, depending on the distribution. Categorical variables were presented as frequencies and percentages. Normality was assessed using the Shapiro–Wilk and Kolmogorov–Smirnov tests. The Mann–Whitney U test was used for variables with abnormal distributions. The Pearson chi-square test was used for 2 × 2 tables with expected counts ≥ 5. Fisher’s exact test was applied to tables with counts < 5. Statistical analyses were performed using StatSoft, Inc. STATISTICA (data analysis software system), version 13. A *p*-value < 0.05 was considered statistically significant.

## 3. Results

**Figure 1:**
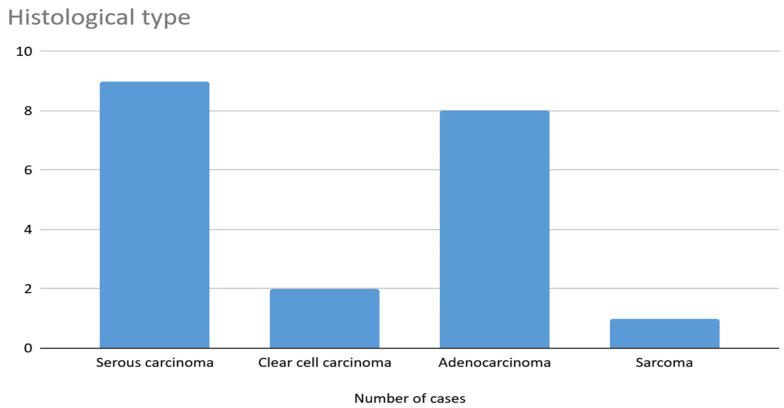
Histological types.

Twenty patients with diagnosed endometrial cancer and a confirmed mutation in the TP53 gene were included in the study; the mean age of the patients was 69.2 years. The exact characteristics of the study group are shown in the Table 1. Patients were classified into two groups depending on the histopathological type of tumor: Group I included patients diagnosed with an endometrial endometrioid tumor and Group II included patients with other histological types. Group I includes nine patients (45%); Group II, 11 patients (50%). The most common histological type in our population was endometrial endometrioid cancer (9 patients, 45%), while the least common type was sarcoma, which was diagnosed in one patient (5%). Detailed data are available in the Figure 1. Four patients (20%) had an associated PIK3CA mutation. In the histological analysis of the malignancy grade, the tumor was found to be G3 in 13 patients and G2 in 7 of them.

No statistically significant difference was found between the groups of qualitative variables, such as BMI or age (*p*-value equal to 0.879 and 0.620, respectively). A box-plot of the BMI comparison is provided below. There was also no significant difference observed in the analysis of the occurrence of pathological genital bleeding at diagnosis (*p* = 0.518). Also, there was no statistically significant difference between the study groups for endometrial thickness determined during transvaginal ultrasound at the time of admission. Details are shown in Table 2.

Also, staging was analyzed according to the 2009 and 2023 FIGO classifications, including mutation status. Details of the classification comparison are shown in Table 3.

**Figure 2:**
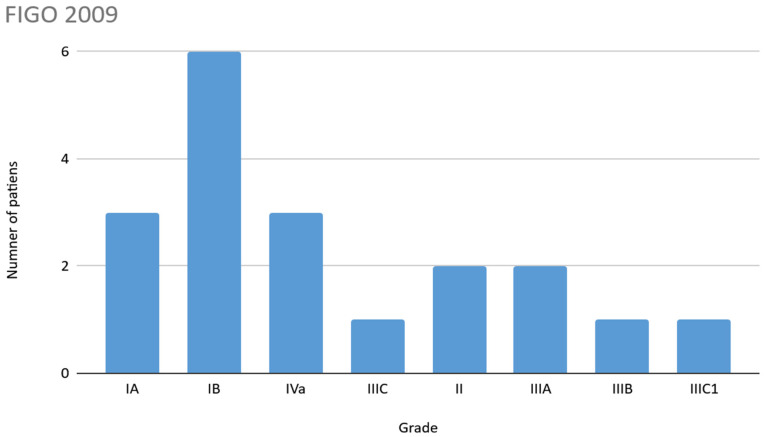
FIGO Classification 2009.

**Figure 3:**
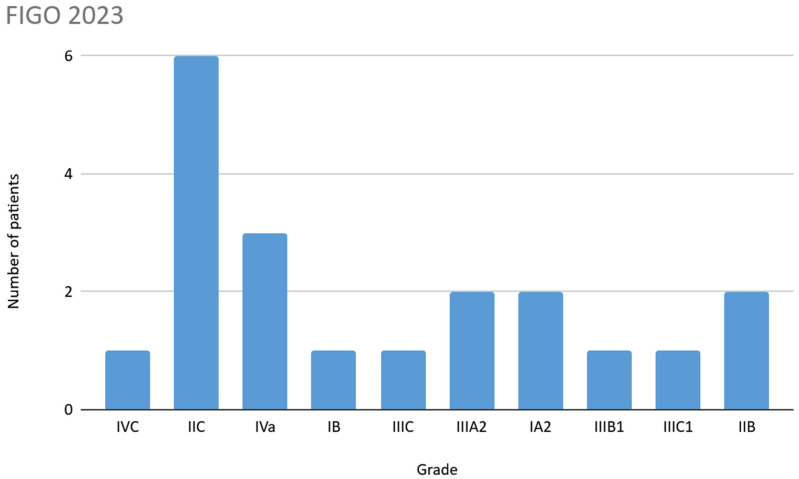
FIGO 2023 classification.

For the FIGO 2009 classification, the most frequent stage was IB, while for the FIGO 2023 classification it was IIC. In 5 patients (20%) a higher stage was observed when using the FIGO 2023 classification; details are shown in Figure 2 and Figure 3. For the FIGO 2023 classification, when taking into account mutations in the TP53 gene, upstaging occurred in 6 patients (30%).

**Figure 4:**
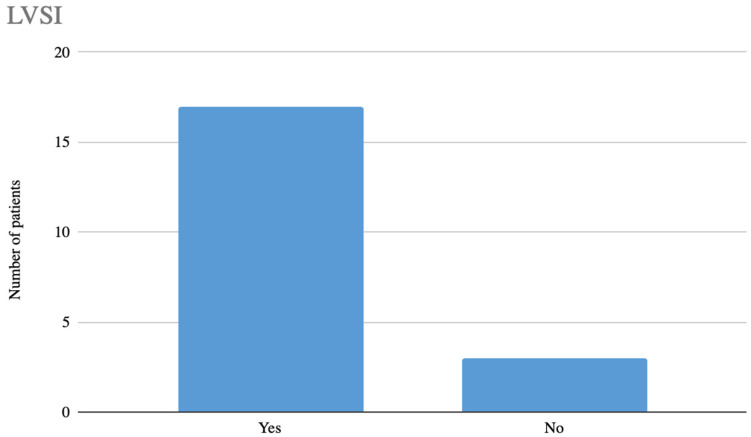
LVSI status in the study population.

Notably, 13 patients (65%) showed features of infiltration of the vascular-lymphatic space (Figure 4). This fact has significant clinical implications and affects the subsequent prognosis of patients. In our patient population, a decision was made to include radiotherapy in 6 of them. Chemotherapy was implemented in 8 patients and radiochemotherapy in 7. In the study cohort, a recurrence was found in one patient. At the time of compiling the results, the longest disease-free survival is 38 months.

## 4. Discussion

Molecular analysis of endometrial cancer with TP53 mutations, including not only the presence of the mutation itself, but also its type, location and coexisting genetic alterations, opens up new perspectives for the identification of therapeutic targets and biomarkers. Statistically, the most common type of TP53 gene mutations are missense mutations, involving single nucleotide substitutions and amino acid changes in the p53 protein. Different types of mutations may be associated with different molecular sequelae. The aforementioned missense mutations lead to the accumulation of mutated p53 protein, which can exhibit oncogenic properties in the cell nucleus. Nonsense mutations or those associated with frameshifts result in a protein with an unstable structure or complete cessation of its production [7]. Currently, in clinical practice, all pathogenic TP53 mutations are grouped in the p53abn category, although there are indications that the specific type and location of the mutation may be clinically relevant, affecting patient prognosis. Further studies correlating specific mutations with response to treatment (e.g., a specific line of chemotherapy, targeted therapies, as well as immunotherapy) are needed, which may allow for even more accurate differentiation of prognosis and choice of therapy in p53abn patients in the future.

The results we obtained from our analysis of a cohort of patients diagnosed with endometrial cancer and p53abn status are mostly consistent with the results available in the literature. It has been shown that patients with this molecular subtype are more likely to present with an advanced stage according to the FIGO classification, which is consistent with our results. In addition, a higher incidence of tumors with high histological malignancy (Grade 3) has been proven [8]. These results are consistent with those obtained in our study, where Grade 3 was found in 54.5% of patients. Similarly, some studies have shown the presence of mutations in older patients [9]. An association between lower BMI and the presence of TP53 mutations has been reported in the literature [7]. Our finding of a high mean BMI (29.5 kg/m^2^) in our cohort notably contrasts with existing literature, which often associates p53-abnormal cancers with a lower BMI compared to their endometrioid counterparts. We propose that this intriguing discrepancy may be influenced by several key factors. Firstly, the high background prevalence of overweight and obesity in the Polish population, where our study was conducted, may act as a significant confounder. Recent epidemiological data indicate that a substantial portion of adults in Poland are overweight or obese, which is a powerful, overarching risk factor for endometrial cancer in general. This high regional prevalence might obscure the more subtle, subtype-specific associations with BMI, leading to a higher mean BMI across all molecular subtypes within our patient population. Secondly, the inherent limitations of a single-center, retrospective study design must be considered. Our institution, as a tertiary referral center, may be subject to selection bias, potentially attracting patients with more complex clinical profiles or multiple comorbidities, including obesity. This could result in a cohort whose risk factor profile is not fully representative of the general population of patients with TP53-mutated endometrial cancer. Therefore, our finding warrants further investigation in larger, multicenter, and prospective studies to disentangle the complex interplay between molecular subtype, BMI, and regional demographic factors.

For patients diagnosed with endometrial cancer and a mutation in the TP53 gene, other molecular alterations often co-occur, representing potential additional therapeutic targets [12]. One of these is ERBB2 (HER2) amplification occurring in 7–19% of patients with p53abn status, regardless of histological type. It leads to overexpression of the HER2 receptor on the surface of cancer cells, which is a direct target for anti-HER2 therapies, e.g., trastuzumab, pertuzumab or antibody–drug conjugates such as trastuzumab-derutexan. The NCT01367002 study demonstrated the benefit of adding trastuzumab to chemotherapy in patients with advanced endometrial cancer [13]. Studies evaluating the efficacy of combinations with newer anti-HER2 drugs are currently underway. The UTOLA study evaluating the use of a PARP inhibitor (Olaparib) for maintenance treatment in patients with advanced endometrial cancer did not show a benefit in the general population, although an exploratory analysis indicated a potential benefit in a subgroup of patients with a homologous recombination deficit [14]. The CANSTAMP study is evaluating the use of niraparib in patients with advanced endometrial cancer and p53abn status as maintenance treatment [15]. The heterogeneity of treatment response observed in studies—for example, the benefit of PARP inhibitors in the HRD group or HER2 inhibitors in the case of amplification—suggests that mutation in the TP53 gene alone is not the only determinant of response to targeted therapies. Consequently, strategies based on multiple biomarkers are needed in the design of future clinical trials. Studies targeting the p53abn subgroup should also include concomitant assessment of coexisting biomarkers to select patients, which will allow the identification of subgroups that benefit most from specific targeted therapies [16].

Lymphatic-vascular space infiltration (LVSI) occurs in a minority of endometrial cancer (EC) cases, and the extent of LVSI is a significant risk factor for disease recurrence and/or metastases. Among patients participating in the PORTEC-1 and PORTEC-2 studies who did not receive radiotherapy, the 5-year risk of recurrence of pelvic lymph node metastases was the higher the more vessels were involved. The PORTEC-3 study provided evidence of the benefit of adjuvant chemotherapy in a subgroup of p53abn patients [17]. Clinical studies making full use of molecular classification to guide therapy are currently underway. Examples include RAINBO or the PORTEC-4a study [18]. In these studies, the heterogeneity of treatment response among p53abn patients is being assessed to allow individualized treatment strategies for patients with p53abn status.

The strengths of our publication include the inclusion of a group of patients with diagnosed endometrial cancer and molecularly confirmed tp53abn status. Gathering data on demographics, co-morbidities and, above all, histological type and FIGO stage provides an opportunity for correlative studies between the factors mentioned. The cohort presented here includes patients with a baseline poorer prognosis due to their TP53 gene mutation status, creating the opportunity to search for further molecular prognostic or predictive factors for targeted therapies. Another advantage is the use of advanced molecular methodology to assess EPCAM genes, i.e., MLH1, MSH2, MSH6, PIK3CA, PMS2, POLE and TP53, which allows parallel and sensitive detection in selected clinically relevant genes.

We acknowledge that the primary limitation of our study is the small sample size of 20 patients drawn from a single center. This inherently limits the statistical power of our analysis and constrains the ability to perform meaningful subgroup analyses, such as in the four patients identified with coexisting PIK3CA mutations. Due to these factors, our findings are susceptible to generalizability bias and should be considered preliminary. Therefore, as we state in our conclusion, we strongly advocate for future multicenter collaborations to expand the cohort, validate these initial observations, and establish more definitive conclusions regarding the clinicopathological features of this high-risk cancer subtype. Furthermore, a significant limitation of the current study is its design, which focused exclusively on a TP53-mutated cohort without a concurrent control group. Therefore, a critical and planned next step for our research group is to conduct a larger, comparative study. Such an analysis will allow us to truly contextualize the unique characteristics of this high-risk subtype against a TP53-wildtype baseline and better isolate the prognostic impact of the TP53 mutation itself. A limitation of the present study is the absence of mature survival data, such as Overall Survival (OS) and Disease-Free Survival (DFS). This is primarily due to the recent timeframe of the patient cohort, which includes individuals treated between January 2023 and March 2025. As a result, the follow-up period is currently insufficient for a robust statistical analysis of long-term outcomes. While we noted that at the time of data compilation, the longest disease-free survival was 38 months and one recurrence had been found, we recognize that this is not enough for comprehensive survival analysis. Given that TP53 mutation status is one of the strongest predictors of unfavorable prognosis, a long-term follow-up study is planned for this cohort. This future analysis will be critical to correlate our clinicopathological findings with definitive survival outcomes and to fully understand the clinical trajectory of these high-risk patients. Another limitation of this study is the absence of data on HER2 and Homologous Recombination Deficiency (HRD) status. These are increasingly crucial biomarkers in TP53-mutated endometrial cancer, guiding targeted therapies such as anti-HER2 agents and PARP inhibitors. The NGS panel utilized in our analysis did not assess for ERBB2 amplification or HRD, precluding the evaluation of these actionable targets in our cohort. We therefore strongly advocate that future research incorporates comprehensive molecular profiling—including p53, MMR, POLE, HER2, and HRD—to fully characterize these high-risk tumors and personalize patient treatment. It should also be noted that with a relatively small group and many potentially related factors (age, BMI, presence of abnormal bleeding, FIGO grade, histopathological classification), there is a risk that the observed correlations will be the result of confounding factors rather than a direct result of cause-and-effect relationships. Another factor to mention is the potential subjectivity in histopathological assessment (observer bias) and inaccuracies in reporting clinical data (reporting bias).

In conclusion, our study presents significant value due to the presentation of a clinically relevant cohort of patients and the availability of clinical data, which creates the potential for further research to identify new therapeutic targets and predictive biomarkers. We would like to highlight the fact that our study is primarily a detailed analysis of a cohort of patients with endometrial cancer and tp53abn status from a clinicopathological perspective. It needs to be extended to a larger group of patients to include other centers in the study and to complete a control group.

## 5. Conclusions

TP53 mutation status allows for the identification of high-risk patients with unfavorable clinicopathological features and a worse prognosis compared to other molecular subtypes, and translates into clinical practice as part of a risk stratification system, influencing decisions on the intensity of adjuvant treatment.

## Figures and Tables

**Table 1:** Characteristics of the study group.

Characteristics	Percentage (%), Number of Patients (N), or Mean (SD)
Total, No.	20
Age, (y)	69.2 ± 9.4
BMI	29.5 ± 10.3
Smokers	3 (15%)
Clinical characteristics	
Endometrial Thickness	24.4 mm—MEAN
Presence of abnormal vaginal bleeding	N = 13. 65%
FIGO Classification (2023)	
IICm	N = 13. 65%
IIIB	N = 2. 10%
IIIC	N = 10. 10%
IVA	N = 1. 5%
IVB	N = 1. 5%
IVC	N = 1. 5%
Histological type	
Endometrioid	N = 9. 45%
Serous	N = 8. 40%
Clear cell carcinoma	N = 2. 10%
Sarcoma	N = 1. 5%

**Table 2:** Comparison of BMI and endometrial thickness between histological subgroups.

Characteristic	Group I (Endometrioid, n = 9)	Group II (Other Types, n = 11)	*p*-Value
BMI (kg/m^2^)			0.879
Min	16.4	18.9	
Max	38.6	62	
Mean	28.3	30.4	
SD	7.3	12.7	
Endometrial Thickness (mm), Mean (SD)			0.62
Min	9	4	
Max	46	34	
Mean	27.8	21.6	
SD	20.6	8.9	

**Table 3:** Comparison of patient populations across FIGO classifications.

FIGO 2009	FIGO 2023	FIGO 2023 M
IVB	IVC	
IA	IIC	IICmp53abn
IB	IIC	IICmp53abn
IVA	IVA	
IVa	IVa	
IVa	IVa	
IB	IB	IICmp53abn
IIIC	IIIC	
II	IIC	IICmp53abn
IIIA	IIIA2	
IA	IA2	IICmp53abn
IIIB	IIIB1	
IIIC1	IIIC1	
IB	IIC	IICmp53abn
IA	IA2	IICmp53abn
II	IIB	
IB	IIC	IICmp53abn
IIIA	IIIA2	
IB	IIB	IICmp53abn
IB	IIC	IICmp53abn

## Data Availability

The data that support the findings of this study are available from the corresponding author upon reasonable request. Due to ethical restrictions, access to the raw data is limited to qualified researchers and may require a data use agreement.

## Abbreviations

The following abbreviations are used in this manuscript:

POLEmut	POLE-ultramutated
MSi-H	Microsatellite Instability-High
LVSI	lymphovascular space invasion
TVUS	transvaginal ultrasound
